# *Experiments on Human Calculi. In a Letter from Mr.* Timothy Lane, *F. R. S. to* William Pitcairn, *M.D. F.R.S.*

**Published:** 1792

**Authors:** 


XIII. *Experiments on Human Calculi. In a Letter from Mr.* Timothy Lane, *F. R. S. to* William Pitcairn, *M. D. F. R. S.*—Vide *Philosophical Transactions of the Royal Society of London, Vol.* LXXXI. *for the Year* 1791. *Part* II. 4to. London, 1791.

THE lixivium saponarium of the late London Pharmacopœia, prepared with the addition of so much lime as nearly to free the salt of tartar of its fixed air *, having been used as a medicine for the stone and gravel, and its effects found very unequal, Mr. Lane was induced, twenty years ago, to examine different calculi, both as to the effect of the above lixivium and of fire upon them.

Great disparity was observed, some being dissolved, and others scarcely altered in their figure.

When tried by fire, some, we are told, were nearly evaporated by a red heat, while others retained their form.

Different parts even of the same calculus, he observes, varied considerably.

* See Letter to Dr. Heberden, Medical Transactions, Vol. I. p. 112.

For

For the ſake of greater accuracy the experiments were carefully repeated; and for this purpoſe fourteen ſpecimens were collected, ſome of which were of the ſame calculus, and others different calculi.

In the experiments by fire our author was favoured with the aſſiſtance of Mr. Staneſby Alchorne, of the Tower, to whom were ſent ten grains of each ſpecimen, in ſeparate papers, which were numbered.

The contents of each paper were placed in ſeparate cupels, under a muffle, the ſame as is uſed by him for aſſaying gold and ſilver. The fire was raiſed gradually till the furnace was fully heated: the time from raiſing the fire to the taking them out again was three hours, when it was concluded that whatever volatile matter they contained was expelled.

The ſame quantity, as above mentioned, of each ſpecimen being put into ſeparate numbered phials, with one ounce meaſure of the lixivium in each, continued forty-eight hours, the phials being frequently ſhaken to forward the ſolution.

The clear liquor of each phial was decanted into freſh phials, and a quarter of an ounce more lixivium was added to ſuch of the ſpecimens mens as were undiſſolved; after twenty-four hours they were poured out of the phials into ſeparate filtering papers, each numbered, and the phials waſhed with diſtilled water, which was alſo poured into the papers, ſo that all that remained undiſſolved might be detained by the papers, which, with their contents, were carefully dried.

The following table ſhows the remains of each:

| | Unſublimed. | Undiſſolved. |
|---|---|---|
| | Grains. | Grains. |
| N° 1. | 1½ | ¾ |
| 2. | 2½ | 2 |
| 3. | ¼ | ¼ |
| 4. | 1¼ | 2 |
| 5. | ¼ | 0 |
| 6. | 3½ | 2½ |
| 7. | 3¼ | 6 |
| 8. | 6 | 8¼ |
| 9. | 6¼ | 6¼ |
| 10. | 6¼ | 7½ |
| 11. | ¼ | ½ |
| 12. | ¼ | 0 |
| 13. | 5½ | 4 |
| 14. | 6 | 5¾ |

The

The appearances of each after calcination were as follows:

N° 1, 3, 7, 8, left a fine white and ſoft powder.

N° 4, 5, 11, 12, left a white and gritty powder.

N° 2, 6, 9, 10, 14, were partly in powder white and gritty, with ſome lumps of a dark colour, as if not fully calcined.

N° 13. Of this the figure, it is obſerved, was not greatly altered; it remained hard, and part of it appeared as if inclined to flux.

After being in the lixivium forty-eight hours,

N° 8, 9, 13, 14, were found ſoft.

N° 7 and 10 remained hard.

Theſe ſix, Mr. Lane obſerves, were ſeparately taken out of the lixivium and put into a mortar, and rubbed or broken, and then carefully returned to their ſeparate phials before the ſecond addition of lixivium, in order to forward the ſolution.

Mr. Lane gives the following deſcription of the different ſpecimens:

N° 1 was the external part of a laminated calculus, of a light yellowiſh brown colour.

The

The nucleus, so called, as being the central part, was, he observes, of a much deeper colour, and had been found not so soluble in lixivium as the light-brown part.

N° 2 was the external part of a calculus, in colour like dirty tobacco-pipe clay.

The nucleus of this, we are told, was of a bright yellow, and more soluble in lixivium than the whitish part.

N° 3 was a light-brown laminated calculus.

N° 4 and 5 were two specimens from one calculus; of which N° 4 was the external coat, of a dirty tobacco-pipe-clay colour.

N° 5 was the internal part of N° 4, yellowish like N° 1.

N° 6 was a calculus taken out of the urethra; of a greyish white, inclining to yellow, and of a porous texture.

N° 7 was a calculus about the size of a nutmeg, taken from a child of a year old; it was ash-coloured, in waves of different shades, laminated and hard.

N° 8 was a dark-brown and very hard calculus, of the mulberry kind.

N° 9 and 10 were two specimens from one calculus; of which N° 9 was the external whitish part, part, which appeared like a coat of calcareous earth, covering an irregular mulberry calculus.

The covering of this calculus induced our author to fufpect that lime or lime-water might have been taken, and, by being decompounded by frefh urine, containing fixed air, have formed this covering. Other calculi, he obferves, have afforded the fame fufpicions.

This circumftance leads him to fuggeft that, in future, an account of medicines taken might, in thefe cafes, afford much information, when joined with the examination of different parts of large calculi taken out of the bladder.

N° 10 was the brown mulberry part covered by N° 9. The three following were parts of one large, laminated calculus; of which

N° 11 was the external lamina, of a brownifh yellow.

N° 12 was the central part, called the nucleus, of a pale orange colour.

N° 13 confifted of fome of the laminæ, between the nucleus and the external coat, of a fparkling appearance.

N° 14 was a whitifh, porous, and eafily broken calculus.

The experiments by fire explain, Mr. Lane thinks, thinks, the different accounts of authors, refpecting the component parts of calculi.

He obferves that, in general, thofe which contained the largeft proportion of volatile parts were moft foluble in lixivium.

The infolubility of fome of them explains, he thinks, the want of fuccefs in feveral cafes, where lixivium, foap, and lime-water, have been given as remedies.

The folubility of others, however, joined with the teftimony of reputable authors, and his own experience for near thirty years, confirm, in his opinion, the falutary effects of lixivium in many cafes.

It frequently happens, he obferves, that, in fits of the gravel and ftone, gravel or fmall pieces of calculi are difcharged, which fhould be examined.

If they are found to be perfectly foluble in lixivium *(aq. kali puri)*, the remedy, he remarks, will be obvious; if imperfectly, doubtful; if infoluble, lixivium will only irritate, without benefit.

